# Preoperative downstaging chemoradiation with concurrent irinotecan and capecitabine in MRI-defined locally advanced rectal cancer: a phase I trial (NWCOG-2)

**DOI:** 10.1038/sj.bjc.6605258

**Published:** 2009-08-18

**Authors:** S W Gollins, S Myint, S Susnerwala, B Haylock, M Wise, C Topham, L Samuel, R Swindell, J Morris, L Mason, E Levine

**Affiliations:** 1 Department of Clinical Oncology, North Wales Cancer Treatment Centre, Rhyl LL18 5UJ, UK; 2 Department of Clinical Oncology, Clatterbridge Hospital, Liverpool CH63 4JY, UK; 3 Department of Clinical Oncology, Royal Preston Hospital, Preston PR2 9HT, UK; 4 Department of Clinical Oncology, St Lukes Cancer Centre, Guildford GU2 7XX, UK; 5 Department of Clinical Oncology, Aberdeen Royal Infirmary, Aberdeen AB25 2ZB, UK; 6 Department of Medical Statistics, The Christie Hospital NHS Trust, Manchester M20 4BX, UK; 7 Department of Clinical Oncology, The Christie Hospital NHS Trust, Manchester M20 4BX, UK

**Keywords:** rectal cancer, MRI staging, neoadjuvant chemoradiation, capecitabine, irinotecan

## Abstract

**Background:**

The aim of this study was to investigate the safety of neoadjuvant chemoradiation using radiotherapy (RT) combined with concurrent capecitabine and irinotecan for locally advanced rectal cancer before surgery.

**Methods:**

Forty-six patients were recruited and treated on the basis that MRI scanning had shown poor-risk tumours with threatening (⩽1 mm) or involvement of the mesorectal fascia. Conformal RT was given using 3 or 4 fields at daily fractions of 1.8 Gy on 5 days per week to a total dose of 45 Gy. Concurrently oral capecitabine was given twice daily throughout radiotherapy continuously from days 1 to 35 and intravenous irinotecan was given once per week during weeks 1 to 4 of RT. Dose levels were gradually escalated as follows. Dose level 1: capecitabine 650 mg m^−2^ b.i.d. and irinotecan 50 mg m^−2^; Dose level 2: capecitabine 650 mg m^−2^ b.i.d. and irinotecan 60 mg m^−2^; Dose level 3: capecitabine 825 mg m^−2^ b.i.d. and irinotecan 60 mg m^2^; Dose level 4: capecitabine 825 mg m^−2^ b.i.d. and irinotecan 70 mg m^−2^.

**Results:**

Diarrhoea (grade 3, no grade 4) was the main serious acute toxicity with lesser degrees of fatigue, neutropenia, anorexia and palmar-plantar erythrodysesthesia. The recommended dose for future study was dose level 2 at which 3 of 14 patients (21%) developed grade 3 diarrhoea. Postoperative complications included seven pelvic or wound infections and two anastomotic and two perineal wound dehiscences. There were no deaths in the first 30 days postoperatively. Of 41 resected specimens, 11 (27%) showed a pathological complete response (pCR) and five (12%) showed an involved circumferential resection margin (defined as ⩽1 mm). The 3-year disease-free survival (intent-to-treat) was 53.2%.

**Conclusion:**

In patients with poor-risk MRI-defined locally advanced rectal cancer threatening or involving the mesorectal fascia, preoperative chemoradiation based on RT at 45 Gy in 25 daily fractions over 5 weeks with continuous daily oral capecitabine at 650 mg m^−2^ b.i.d. days 1–35 and weekly IV irinotecan at 60 mg m^−2^ weeks 1–4, provides acceptable acute toxicity and postoperative morbidity with encouraging response and curative resection rates.

The mainstay of curative treatment for rectal cancer is surgery and the surgical technique of total mesorectal excision (TME), wherein meticulous sharp dissection is carried out in the plane of the mesorectal fascia, has been shown to reduce the chance of local recurrence (MacFarlane *et al*, 1993; Kapiteijn *et al*, 2001). Perioperative RT can reduce the chance of local recurrence following rectal cancer surgery (Colorectal Collaborative Group, 2001). The FFCD 9203 and EORTC 22921 trials have shown that for operable rectal cancer combined fluoropyrimidine chemotherapy and long-course RT confers an advantage in terms of reducing local pelvic recurrence compared with RT alone (Bosset *et al*, 2006; Gerard *et al*, 2006). A large randomised trial has definitively shown that preoperative chemoradiation (CRT) reduces local recurrence in operable rectal cancer compared with postoperative CRT (13 *vs* 6%), with reduced acute and late toxicity (Sauer *et al*, 2004).

Involvement of the circumferential resection margin (CRM) has been shown to be an important, independent prognostic factor, resulting in high rates of local recurrence (Quirke *et al*, 1986; Wibe *et al*, 2002), distant metastases (Hall *et al*, 1998; Mawdsley *et al*, 2005) and worse survival (Birbeck *et al*, 2002; Nagtegaal and Quirke, 2008), even after TME surgery (Marijnen *et al*, 2003).

Several potential mechanisms have been described in which the histological CRM may be positive including direct or discontinuous tumour spread, lymph node spread, lymphovascular spread and perineural spread (Nagtegaal and Quirke, 2008). CRM positivity is also strongly related to the quality of surgery (Nagtegaal *et al*, 2002; Quirke *et al*, 2009).

In the United Kingdom and increasingly elsewhere, preoperative MRI scanning of the pelvis is viewed as the gold standard for judging threatening or involvement of the mesorectal fascial potential surgical TME resection plane. In 311 patients with operable rectal cancer who were shortly due to undergo surgery, the MERCURY trial showed that preoperative MRI has high accuracy, negative predictive value and specificity in the identification of a potentially involved CRM (MERCURY Study Group, 2006).

Approximately 20–30% of rectal cancer patients present with disease which threatens or involves the potential mesorectal surgical excision margin. These patients might be considered to have ‘locally advanced inoperable’ disease with a high risk of local recurrence unless some form of downstaging is carried out before surgery. As an indication of what might be achieved in the modern era using downstaging CRT with a single agent fluoropyrimidine as a radiation sensitiser in patients with locally advanced carcinomas, a single UK cancer network audit has been reported in 150 patients with locally advanced rectal cancer (Mawdsley *et al*, 2005). Sixty-one percent were T4 and approximately one-third had been staged by MRI. Concurrent bolus 5-FU was administered in weeks 1 and 5 of radiotherapy as per the EORTC 22921 and FFCD 9203 trials. On an intention to treat (ITT) analysis, an R0 resection was achieved in only 65% of patients and the poor prognostic significance of involved CRM post-downstaging CRT was confirmed with 3-year local recurrence at 10 *vs* 62% and 3-year disease-free survival at 52 *vs* 9% for R0 *vs* R1/2 resections, respectively.

There is a need to improve on the potency of currently available downstaging CRT regimes and one potential way of achieving this is by adding in a second chemotherapy agent to form a doublet in addition to a fluoropyrimidine. The topoisomerase-I inhibitor irinotecan is one possible agent to use in this context in view of radiosensitising properties demonstrated *in vitro* (Chen *et al*, 1997). Several early phase studies have been reported (Mehta *et al*, 2003; Klautke *et al*, 2005; Mohiuddin *et al*, 2006; Navarro *et al*, 2006; Glynne-Jones *et al*, 2007) including by our own group (Iles *et al*, 2008) concerning rectal cancer CRT regimes using the doublet of 5FU plus irinotecan as radiation sensitisers. Rates of grade 3 or 4 toxicity have varied from between 12–37% (mostly diarrhoea) with promising pathological complete response (pCR) rates of between 14–37% for resected specimens.

There are potential advantages in using the oral 5FU prodrug capecitabine rather than 5FU itself including the avoidance of a central venous catheter to administer continuous infusional 5FU. In addition, the final enzymatic conversion of capecitabine is mediated via thymidine phosphorylase which often occurs in a higher concentration in cancer tissue compared with adjacent normal tissue with the potential advantage of improving the therapeutic ratio compared with 5FU (Glynne-Jones *et al*, 2006). Thymidine phosphorylase is upregulated by RT (Sawada *et al*, 1999).

This study represents a progression of previous research reported by our group (the North West/North Wales Clinical Oncology Group) which used irinotecan and infusional 5FU as a doublet of concurrent radiation sensitisers in the preoperative downstaging of locally advanced rectal cancer (Iles *et al*, 2008). This study is a phase I trial in patients with MRI-defined locally advanced rectal cancer which is threatening or involving the mesorectal fascia and replaces the infusional 5FU with continuous oral capecitabine. The doses of irinotecan and capecitabine were gradually increased, keeping the radiation dose constant, with toxicity being the primary end point.

## Patients and methods

### Inclusion criteria

Male or female patients aged ⩾18 years old, of WHO performance status 0, 1 or 2 were included. All had provided written informed consent to participate in the trial. Patients had histologically confirmed previously untreated carcinoma of the rectum with the distal extent within 12 cm of the anal verge using a rigid sigmoidoscope. They were deemed to be a candidate for preoperative downstaging chemoradiation due to T3 disease on MRI scanning ⩽1 mm from the edge of the mesorectum or T4 disease on MRI scanning or any T3/T4 disease on MRI scanning with the distal extent of tumour ⩽5 cm from the anal margin. Any nodal status (N0-2) was permitted and computerised tomography (CT) scanning of abdomen and pelvis had failed to detect evidence of metastatic (M1) disease. All radiological investigations were to be carried out within 4 weeks of trial registration. Patients had adequate haematology with a neutrophil count >1.5 × 10^9^ l^−1^, platelet count >100 × 10^9^ l^−1^, Hb>9 g dl^−1^ (the use of blood transfusions was allowed to increase the level of haemoglobin). Patients also had adequate renal and hepatic function with serum creatinine ⩽1.5 × ULN, serum bilirubin ⩽1.25 × ULN, serum ALT, AST and alkaline phosphatase ⩽2.5 × ULN.

### Exclusion criteria

Any of the following was regarded as a criterion for exclusion from the trial: previous systemic chemotherapy; previous radiotherapy to the planned exposure area; any severe concurrent medical condition which would make it undesirable, in the supervising clinician's opinion, for the patient to participate in the trial or which would jeopardise compliance with the trial protocol; a calculated creatinine clearance of less than 50 ml min^−1^; loss of continuity of the upper GI tract or malabsorption; a history of myocardial infarction within previous year and/or with unstable angina, arrythmia or cardiac failure; pregnancy or lactation; patients of child-bearing potential not implementing adequate contraception; previous or current malignancies at other sites, with the exception of adequately treated *in situ* carcinoma of the cervix uteri and basal or squamous cell carcinoma of the skin; subjects considered by the investigator to be at risk of transmitting any infection through blood or other body fluid including acquired immune deficiency syndrome, or other sexually transmitted disease or hepatitis; participation in other clinical trials; partial or complete bowel obstruction (though patients in whom this had been relieved with a defunctioning stoma, were permitted to enter the trial).

### Study structure

The primary objective of the study was to determine the maximum-tolerated dose (MTD) of intravenous irinotecan and capecitabine when given concurrently with long-course preoperative pelvic radiotherapy in patients who have locally advanced rectal cancer. The regime defined using the MTD of capecitabine and irinotecan could then be taken forward into a future phase II trial.

Patients were treated with a 5-week course of downstaging CRT. The dose of radiotherapy was fixed at 45 Gy and the aim was to gradually increase the doses of capecitabine and irinotecan in cohorts of three patients until the MTD had been defined. An MRI scan was carried out 6 weeks following CRT and surgery attempted 2 weeks following the MRI scan, that is, 8 weeks following completion of CRT. Acute toxicity was assessed weekly throughout the 5-week course of CRT then weekly for 4 weeks afterwards. Postoperative morbidity was assessed up to 30 days post surgery. Late toxicity was assessed at 6, 12, 24 and 36 months post completion of CRT.

### Radiotherapy

RT was planned with patients in the prone position with a full bladder (depending on patient tolerance) and an anal marker. Either CT or fluoroscopic simulation (with rectal barium contrast) were acceptable for planning RT. Opacification of the small bowel was recommended (e.g., 300 ml Baritop plus 20 ml Gastrograffin orally 45–60 min before simulation). A belly board was not routinely used.

A Gross Tumour Volume (GTV) was defined using clinical evidence and pelvic radiological imaging. The Planned Target Volume (PTV) was defined as follows. Superiorly 3 cm superior to the most superior extent of the GTV, but PTV to extend no higher than the sacral promontory. Inferiorly 3 cm inferior to the most inferior extent of GTV. Posteriorly the border of the most posterior aspect of sacrum. Anteriorly 2 cm anterior to tumour or the anterior rectal wall whichever is the more anterior. Laterally 3 cm lateral to the most lateral extent of GTV.

RT was prescribed to the central axis of the beams. In the central axis section, the dose within the target area was stipulated to be no less than 95% and no more than 105% of the prescribed dose. Treatment was to 45 Gy in 25 daily fractions over 5 weeks, treating for 5 days (Monday–Friday) per week at 1.8 Gy per day. Three or four treatment fields were allowed and all fields were to be treated daily.

### Chemotherapy

The haematologic and serum biochemical parameters outlined in ‘Inclusion Criteria’ (above) had to be confirmed within the week before commencing study treatment.

Capecitabine was taken continuously throughout the 5-week course of radiotherapy including weekends. The capecitabine tablets were taken twice daily approximately 12 h apart within 30 min of the ingestion of food (ideally after breakfast and evening meal) with approximately 200 ml of water (not fruit juices), and before radiotherapy on day 1. Irinotecan was given as a 60 min intravenous infusion in 250 ml of normal saline once per week during weeks 1, 2, 3 and 4 of radiotherapy (with equal, weekly spacing between infusions). There was flexibility as to which day in the week was chosen to administer irinotecan.

The starting dose (dose level 1) of capecitabine was 650 mg m^−2^ b.i.d. and irinotecan 50 mg m^−2^. The doses were then gradually escalated as follows: dose level 2: capecitabine 650 mg m^−2^ b.i.d. and irinotecan 60 mg/m^2^; dose level 3: capecitabine 825 mg/m^2^ b.i.d. and irinotecan 60 mg/m^2^; dose level 4: capecitabine 825 mg/m^2^ b.i.d. and irinotecan 70 mg/m^2^.

It was planned that there would be three patients per cohort. Toxicity was assessed until 4 weeks post completion of radiotherapy that is, 9 weeks in total. If no dose-limiting toxicity (DLT) was encountered, further patients would be entered at the next higher dose level. The next dose level was not available to recruitment until toxicity data was available for the whole 9-week period for all patients on a particular dose level. If one patient developed grade 3/4 toxicity, then a further three patients would be added to that cohort. If there were no further episodes of grade 3/4 toxicity (i.e., one of six patients showed a DLT), then further patients would be entered at the next higher dose level. If at least two out of six patients at the same dose level developed DLT, then this was to be considered as too toxic and the next three patients were treated at the dose level lower. The highest dose level at which zero of three or one of six patients developed DLT was considered the MTD. It was the intention to use the CRT regimen at the MTD in a subsequent phase II trial in an expanded number of patients (100).

### Acute toxicity assessment and dose adjustment

Toxicity was scored according to the National Cancer Institute Common Toxicity Criteria version 2.0 (published 30 April 1999). DLT was defined as: grade 3 or 4 diarrhoea; grade 3 or 4 fatigue; grade 3 or 4 neutropenia accompanied by fever (>38°C) or ⩾grade 3 infection; grade 4 thrombocytopenia; grade 4 nausea/vomiting despite full antiemetic treatment; grade 3 palmar–plantar erythrodysesthesia (Hand Foot Syndrome); dose delay of >2 weeks because of drug-related toxicity.

If grade 1 haematological toxicity was encountered no adjustment of RT or chemotherapy took place. For grade 2 haematological toxicity no adjustment of radiotherapy took place but chemotherapy was interrupted until grade 0–1 then continued at 100% dose. For grade 3 haematological toxicity RT was interrupted until grade 0–1 then continued and chemotherapy interrupted until grade 0–1 then continued at 75% dose. For grade 4 haematological toxicity RT was interrupted until grade 0–1 then continued and chemotherapy discontinued permanently.

If grade 1 non-haematological toxicity was encountered no adjustment of RT or chemotherapy took place. For grade 2 non-haematological toxicity daily review of RT took place but chemotherapy was interrupted until grade 0 then continued at 100% dose. For grade 3 non-haematological toxicity daily review of RT took place and chemotherapy interrupted until grade 0 then continued at 75% dose. For grade 4 non-haematological toxicity RT was discontinued unless toxicity settled to grade 0–1 within 2 weeks when it could be continued and chemotherapy was discontinued permanently.

### Surgery and histopathological examination of the resected specimen

A defunctioning stoma was permitted before CRT if judged clinically necessary because of intolerable pelvic symptoms. Total mesorectal excision was used for surgical resection. Histopathological examination of surgically resected specimens (including India inking of the CRM) was carried out according to minimum data set guidelines issued by the UK Royal College of Pathologists (Quirke and Williams, 1998).

### Study conduct

The study had full UK Multicentre Research Ethics Committee approval (MREC/04/4/015), Clinical Trials Authorisation from the Medicines and Healthcare Regulatory Agency for the use of irinotecan (MF 8000/12694) and capecitabine (MF 8000/12695) and was overseen by an Independent Data Safety Monitoring Committee. It was conducted according to European Clinical Trials Directive 2001/20/EC and sponsored by Conwy and Denbighshire NHS Trust. It was included on the UK National Cancer Research Network portfolio (NCRN Trial ID: 1387). The trial was conducted in accordance with The Declaration of Helsinki and Good Clinical Practice Guidelines.

### Statistics

Kaplan–Meier censored survival curves were used, plotted with regard to the 46 patients in the intent-to-treat population. Disease-free survival was defined as the first event of local pelvic recurrence, distant metastases or death.

## Results

### Patient characteristics

Between September 2003 and June 2005 a total of 47 patients consented to participation in the trial of which 46 commenced treatment (one patient withdrew consent before starting treatment). Details of patients and tumour characteristics are shown in Table 1. Five patients had a defunctioning stoma before treatment.

### Treatment-related toxicity/dose intensity

Details of toxicity at the various dose levels are illustrated in Table 2. The most common grade 3 toxicity was diarrhoea (recorded overall in 10 patients), Other grade 3 toxicities (some overlapping), which occurred included fatigue (three patients), anorexia (two patients), uncomplicated neutropenia (three patients), neutropenic fever (two patients), nausea/vomiting (two patients), palmar–plantar erythrodysesthesia (PPE) (one patient), abnormal liver enzymes (one patient) and hypertension (one patient).

At dose level 1 there were no dose-limiting toxicities and thus the trial progressed to dose level 2.

One of the first three patients on dose level 2 developed a protocol-stipulated DLT (grade 3 diarrhoea) and thus three additional patients were added on this dose level with no further DLTs. Progression was then made to dose level 3.

Owing to an administrative error, seven patients were initially treated at dose level 3. One of these suffered a DLT (grade 3 fatigue). One other patient, a 72-year-old female, who had been well, presented with an acute neurological event on day 22 of therapy, characterised by almost complete dysphagia, marked drowsiness and nausea suggesting a possible brain stem cerebrovascular accident although this was not confirmed on an MRI scan. No further chemotherapy was given although she went on to complete her RT after an 8-day delay with a subsequent slow neurological recovery over the following 2 months. There was uncertainty as to the nature of this episode, particularly in view of the fact that she did not show other anticipated treatment-related toxicities (no diarrhoea, neutropenia or dehydration) and in view of this progression was then made to dose level 4.

On dose level 4, two of six patients experienced DLT (grade 3 diarrhoea and neutropenic fever) and thus the trial stepped down a dose level back to dose level 3 as the provisional initial recommended dose for expanding out for treatment of an increased number of patients.

Of the first additional 16 patients treated at dose level 3 however, seven developed a DLT. This meant that a total of 8 of 23 patients (34%) treated on dose level 3 had developed at least one DLT (Table 3).

After review of the data by the Independent Data Safety Monitoring Committee, dose level 2 was chosen as the revised recommended dose at which to include further patients. A further eight patients were then treated at dose level 2 of which two developed a DLT (grade 3 diarrhoea). Thus in total, 3 of 14 patients (21%) at dose level 2 developed a DLT, which was considered acceptable as a recommended dose to use in future studies.

**Figure 1:**
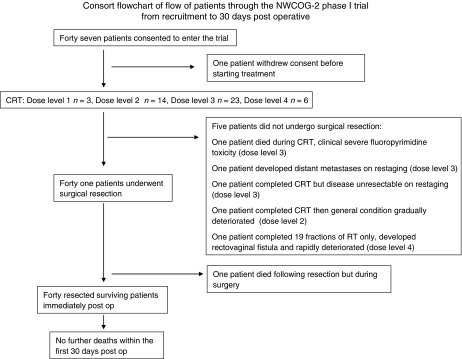
Consort flowchart of flow of patients through the NWCOG-2 phase I trial from recruitment to 30 days post operative.

The consort flowchart showing the flow of patients through the study is shown in Figure 1. Of the 46 patients who commenced treatment, five (11%) did not undergo surgical resection and their details are as follows.

A 68-year-old female patient (dose level 3) had been well until week 4 of treatment, being admitted as an emergency on day 28 of her treatment course. She experienced a rapid onset of diarrhoea and on admission was dehydrated with grade 3 PPE, grade 3 neutropenia, marked mucositis and hypoalbuminaemia. She was treated with full supportive measures including intravenous rehydration and broad spectrum antibiotics but her condition continued to deteriorate, resulting in her death from pneumonia a week post admission. A 59-year-old male patient (dose level 3) developed liver metastases on re-staging. A 73-year-old male patient (dose level 3) completed CRT but was found to have unresectable disease on re-staging. A 65-year-old male patient (dose level 2) completed CRT but suffered a gradual deterioration in general condition, which meant that he was never fit for resection, dying approximately a year later. Finally, a 66-year-old female patient developed a recto-vaginal fistula during CRT, completing only 19 fractions of radiotherapy. She experienced a rapid deterioration in her general condition resulting in death.

One male patient (dose level 3) developed intraoperative bleeding and had a cardiac arrest and died on the operating table, immediately following resection of his cancer.

Overall 5 of 13 females (38%) developed a grade-3 toxicity compared with 8 of 33 males (24%) although this difference was not significant using *χ*^2^ (*P*=0.55).

Dose intensity that was achieved, expressed in terms of the average percentage of the total intended dose that could be delivered at each dose level is shown in Table 4. At all dose levels at least 96% of the intended dose of RT was able to be delivered and at least 93% of the intended dose of irinotecan. However, the achievable dose intensity of capecitabine was lower with only 82% deliverable at dose levels 3 and 4. At the eventual recommended dose level 2, the achievable dose intensities were: RT 100%, irinotecan 96% and capecitabine 91%.

### MRI post chemoradiation

Forty-three patients had a second MRI post CRT. Three patients did not have a second MRI because of death during CRT of one patient, development of a recto-vaginal fistula during CRT in one patient with rapid clinical deterioration then death and patient refusal in a third (subsequently resected with a negative CRM).

Comparing pre- *vs* post-CRT MRI scans (Table 5), 18 patients (42%) were judged to have had their T-stage downstaged, 25 (58%) unchanged and none upstaged. None of nine patients with N0 disease on their pre-CRT MRI scan were upstaged. Of 34 patients who had had N1 or N2 disease on their pre-CRT MRI scan, 30 (88%) were judged to have had their N stage downstaged, four (12%) unchanged and none upstaged.

### Surgery

The median time between the last fraction of RT and surgery was 59 days (range 41–118 days). 41 patients underwent resection: three of three included at dose level 1, 13 of 14 at dose level 2, 20 of 23 at dose level 3 and five of six at dose level 4. Thirty-one patients underwent an anterior resection, nine an abdominoperineal resection and one a Hartman's procedure. Twenty-two of the 31 patients having an anterior resection had a defunctioning stoma (17 ileostomy and five colostomy). Postoperatively the median inpatient stay was 11 days (range, 6–42 days).

The postoperative complications prospectively recorded as occurring within 30 days of surgery are shown in Table 6. Of the 40 patients who underwent resection and were alive postoperatively none died within the 30-day postoperative period. There were 7 pelvic or wound infections and two anastomotic and two perineal wound dehiscences.

### Histology

Of 41 resected specimens, 11 (27%) showed a pathological complete response (pCR; T0), two (5%) were T1, four (10%) T2, 23 (56%) T3 and one (2%) T4. Thirty-three (80%) were N0, five (12%) N1 and three (7%) N2. The average nodal yield was 14 per specimen (range, 2–43). Numbers of patients showing a pCR/number of resected specimens at each dose level were as follows: 1/3 at dose level 1, 6/13 at dose level 2, 3/20 at dose level 3 and 1/5 at dose level 4. Ten patients (24%) had only microfoci of disease remaining (2 T1, 3 T2, 5 T3, 9 N0 and 1 N1).

Overall 36 of the 41 specimens (88%) showed a histologically clear CRM (R0 resection) and five (12%) an involved CRM (defined as ⩽1 mm). Three out of 26 patients whose RT was planned using fluoroscopy were CRM positive compared to 2 of 20 patients who underwent CT planning (*P*=0.76 by *χ*^2^). Two of the five CRM-positive patients were in the low rectum, two mid and one upper and the post-CRT MRI predicted involvement in three of the five cases. Three CRM-positive cases were predominantly anterior and two predominantly posterior.

Comparing pre-CRT MRI scans with histology of the resected specimen (Table 7), 20 patients (49%) had their T-stage downstaged and one (2%) upstaged. Twenty-five of 32 initial MRI N1-2 patients (78%) had their N stage downstaged and all of nine initial MRI N0 patients were N0 on resection.

### Long-term clinical outcome

**Figure 2:**
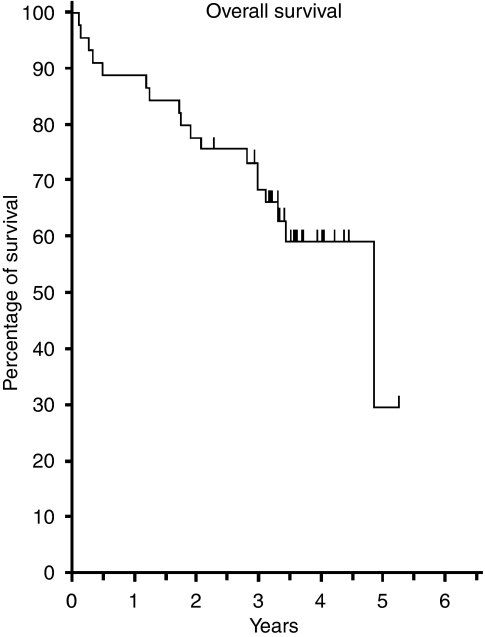
Overall survival (*n*=46).

**Figure 3:**
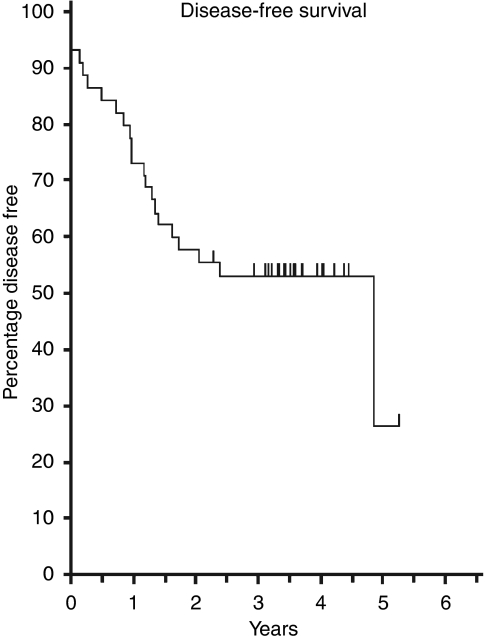
Disease-free survival (*n*=46).

**Figure 4:**
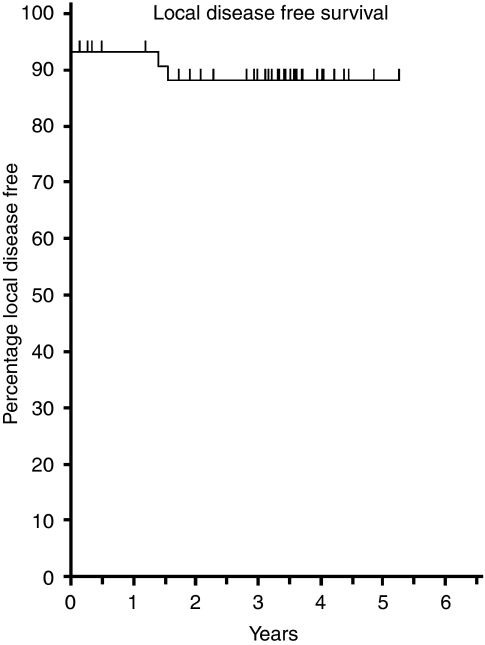
Local pelvic disease-free survival (*n*=46).

**Figure 5:**
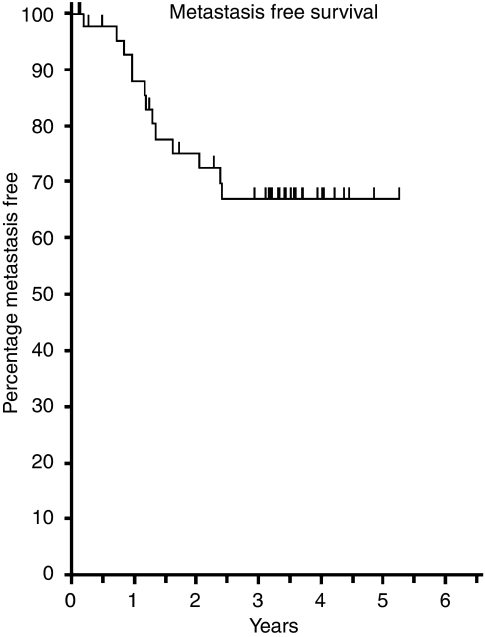
Distant metastasis-free survival (*n*=46).

Although not formally the end point of this phase I trial, the median length of follow-up of patients was 41.2 months, which presented an opportunity to examine longer-term outcomes. For the whole group of 46 patients the 3-year overall survival was 68.5% (Figure 2), disease-free survival 53.2% (Figure 3), pelvic disease-free survival 88.3% (Figure 4) and distant metastasis-free survival 67.0% (Figure 5). Of the 40 patients whose tumour was resected and were alive immediately postoperatively, two developed a local pelvic recurrence at 9 and 13 months post surgery. In all, 13 patients developed distant metastatic disease.

None of the five histologically CRM-positive cases developed locally recurrent disease on follow-up although two developed distant metastases, dying at 24 and 58 months. Of the other three CRM-positive patients, one died at 20 months of other causes and two were alive and well when last seen at 40 and 47 months.

## Discussion

This study is a phase I study examining the combination of irinotecan and capecitabine as concurrent radiation sensitisers in the downstaging CRT of locally advanced rectal cancer before attempting surgery. Among studies examining this CRT regimen this study is unique in using MRI scanning as an inclusion criteria to show threatening or involvement of the mesorectal fascial excision plane by the primary tumour. MRI scanning is now regarded as the investigation of choice in staging rectal cancers with regard to this feature and judgment of operability or not, meaning that rectal cancers included in this study can be more confidently staged as ‘locally advanced inoperable’ than other studies hitherto published using this CRT regimen (Hofheinz *et al*, 2005; Klautke *et al*, 2006, 2007; Willeke *et al*, 2007) (Table 8). These have predominantly used trans-rectal ultrasound (TRUS) and CT scanning for staging, with no requirement for MRI staging. The poor-risk nature of the patients in our study was confirmed by the 53.2% 3-year DFS (Figure 3).

In keeping with the above previously reported studies the predominant DLT encountered in this study was diarrhoea, with lesser degrees of fatigue, neutropenic sepsis, anorexia and PPE. No patient developed grade 4 diarrhoea and at our recommended dose level 2 of capecitabine 650 mg m^−2^ b.i.d. days 1–35 and irinotecan 60 mg m^–2^ once per week during weeks 1, 2, 3 and 4 combined with an RT dose of 45 Gy in 25 daily fractions over 5 weeks, we recorded acceptable acute toxicity with 3 of 14 patients (21%) developing grade 3 diarrhoea.

In the study by Hofheinz *et al* (2005) the recommended dose was irinotecan at 50 mg m^−2^ once per week for five consecutive weeks (250 mg m^−2^ total) plus capecitabine at 500 mg m^−2^ b.i.d. 7 days per week on days 1–38 throughout a course of RT delivering 50.4 Gy in 1.8 Gy fractions over 5.5 weeks. Only one of 12 patients developed a serious toxicity (grade 3 fatigue). However, when the dose of capecitabine was increased to 625 mg m^−2^ b.i.d., three of seven patients developed grade 3 diarrhoea, one grade three nausea and one grade three anorexia.

Willeke *et al* (2007) treated 36 patients with a similar regimen to the recommended dose of Hofheinz *et al* (2005). Again, they found a relatively low rate of grade 3 diarrhoea (11%) and fatigue (3%) although 25% of patients had grade 3 or 4 leukopenia.

The recommended dose in the study by Klautke *et al* (2006) delivered an identical overall irinotecan dose to that in this study (240 mg m^−2^ total) although this was delivered in 6 rather than 4 weekly treatments. The recommended daily capecitabine dose at 750 mg m^−2^ b.i.d. was higher and delivered for a week longer (continuously days 1–43) than that in this study. The radiation dose was also 24% higher at 55.8 Gy delivered in 31 daily fractions over 6 weeks. At this dose level, however, 6 of 16 patients (38%) developed grade 3 diarrhoea.

In view of the above, Klautke *et al* (2007) modified their regimen, maintaining the same daily dose of capecitabine at 750 mg m^−2^ b.i.d. but giving this for a total of 4 weeks only (weeks 1, 2, 4 and 5) rather than for 6 weeks. In addition the total dose of irinotecan delivered was reduced to 200 mg m^−2^, given in the same 4 weeks in which capecitabine was delivered (weeks 1, 2, 4 and 5). In 20 patients this was well tolerated with 10% grade 3 diarrhoea but no histological specimen showed a pCR. This then prompted the use of an increased total irinotecan dose (to 240 mg m^−2^, in four treatment weeks 1, 2, 4 and 5) in a further 20 patients. The rate of grade 3 diarrhoea increased slightly (to 15%) but seven specimens (35%) now showed a pCR.

This study differs from the above initially in delivering a lower dose of 45 Gy of RT rather than 50.4–55.8 Gy. In addition, smaller volumes of tissue were irradiated in this study compared with others with the maximum superior extent of the PTV at the sacral promontory. It is likely that the predominant acute toxicity seen of diarrhoea is largely caused by small bowel enteritis, partly because of the chemotherapy element but also partly because of small bowel in the radiation field. Klautke *et al* (2006) stipulated that the superior border of the radiation field included the fifth lumbar vertebral body and Hofheinz *et al* (2005) and Willeke *et al* (2007) stipulated that the upper border of the clinical target volume (CTV) was at L4–5 for cN-positive patients and the lower border at 5 cm below macroscopic tumour. It is noteworthy that the superior field border had been reduced to the L5/S1 junction in the later report of Klautke *et al* (2007), possibly contributing to the reported reduction in acute toxicity.

We gave capecitabine continuously throughout RT including weekends to mimic the continuous infusion 5FU that was used in our previous study (Iles *et al*, 2008). At the recommended dose level 2 in this study the overall dose of capecitabine delivered was 45 500 mg m^−2^ compared to 38 000 mg m^−2^ in Hofheinz *et al* (2005) and Willeke *et al* (2007) and 64 500 mg m^−2^ in Klautke *et al* (2006). We found that the component of our CRT regimen that was difficult to deliver in its entirety because of toxicity, as the dose levels were increased was the capecitabine. At dose level 3 at which a total dose of capecitabine of 57 750 mg m^−2^ was intended to be given, a mean of only 82% of the intended dose could be delivered (Table 4). It is striking that Klautke *et al* (2007) needed to modify their capecitabine to achieve acceptable toxicity. Whether this was due to the two enforced weekly breaks or simply due to a reduction in the overall delivered dose to 42 000 mg m^−2^ is unclear.

In contrast to the other studies mentioned above, we adopted a strategy of giving irinotecan weekly for the first 4 weeks of a 5-week course of rectal CRT. This approach was similar to that used in our previous study when irinotecan was combined with continuous infusion 5FU throughout the course of RT (Iles *et al*, 2008). The rationale for this was to ‘front-load’ the irinotecan so that a dose would not be delivered in the final fifth week when diarrhoea was liable to be approaching its maximum. We found that at all dose levels a mean of at least 93% of the intended dose of irinotecan could be delivered.

Although not the primary end point of this study, the pCR rate of 27% in resected patients (or 24% by ITT) is promising and lies within the range of 15–35% reported by others (Table 8). The rate of histologically clear surgical CRM reported in this study (88% of resected specimens or 78% by ITT) is also encouraging, as is the low pelvic recurrence rate (Figure 4).

It is likely that the toxicity and tumour response resulting from the combination of a doublet of chemotherapy in addition to RT are complex and dynamic processes with potential marked resultant differences depending on the interplay of the chemotherapy administration schedule and dose and the RT volume, timing, dose and fractionation. The CRT regime (dose level 2) recommended for taking forward into further studies in the current report, would not be out of keeping with those recommended in the other studies although there are significant differences as outlined above. It may appear counter-intuitive to combine three agents (RT, capecitabine, irinotecan) all of which have the overlapping toxicity of diarrhoea although it does appear that at the doses recommended in this study the incidence of this toxicity at serious levels is acceptable. Indeed, at the doses recommended by ourselves and others, rates of diarrhoea reported are not dissimilar to those reported using the doublet of capecitabine plus oxaliplatin as radiation sensitisers in this context (Glynne-Jones *et al*, 2006).

The patient who died in this study on dose level 3 showed signs of severe fluoropyrimidine toxicity. It may be that this patient had a specific metabolic disorder such as dihydropyrimidine dehydrogenase deficiency increasing susceptibility to fluoropyrimidine toxicity although no specific testing for this was carried out. It does, however, emphasise the importance of giving patients on oral fluoropyrimidines clear advice that if they become unwell or show signs of fluoropyrimidine toxicity, especially if these are developing rapidly, then they must stop their tablets immediately and ring promptly for medical advice. For patients receiving aggressive treatment with a CRT regime such as that discussed in this report, this is especially important.

When considering downstaging CRT for rectal cancer, toxicity not only during CRT is important, but also expressed as postoperative morbidity. Hofheinz *et al* (2005) reported that of 19 resected patients, nine (47%) experienced a variety of postoperative complications including wound dehiscence, bowel atonia, bladder dysfunction, recto-vaginal fistula, a presacral abscess needing drainage and anastomotic insufficiency and complicated secondary wound healing requiring revision. There were no intra- or postoperative deaths.

Willeke *et al* (2007), using a similar regimen to Hofheinz *et al* (2005), reported that of the 34 operated patients nine (26%) had prolonged or complicated wound healing, eight (24%) had temporary bowel atonia, three (12%) developed an anastomotic leakage and three (12%) an abscess. Two patients died postoperatively from septic complications.

Klautke *et al* (2006) reported that of 25 operated patients there was one anastomotic leakage and one bowel atonia treated conservatively. There were, however, two deaths, one from pneumonia and the other from sudden cardiac death. In their later report (Klautke *et al*, 2007) no detailed report of postoperative complications is presented other than to state that at the modified dose discussed above, there were no postoperative deaths.

In this study there was one death due to intraoperative bleeding and a consequent cardiac arrest in a patient on the operating table who had had their rectal cancer resected. No cause for this was found. In the 40 patients who underwent resection and were alive postoperatively there were no deaths within the first 30 days and postoperative morbidity (Table 6) did not appear to be unduly severe.

In conclusion, we have developed a CRT regime for use in the preoperative downstaging of locally advanced rectal cancer incorporating the chemotherapy doublet of capecitabine and irinotecan. This regime needs to be included in future studies in larger numbers of patients to determine whether any advantage is conferred compared with the use of a single agent fluoropyrimidine as a radiation sensitiser in this context.

## Figures and Tables

**Table 1:** Baseline patient and tumour characteristics

**Gender**	**Number (%)**
Male	33 (72)
Female	13 (28)
	
**WHO PS**	**Number of patients (%)**
0	37 (80)
1	8 (17)
2	1 (2)
	
Age: median 61.5 years (range, 34–78 years)	
	
Distance from distal tumour margin to anal verge using rigid sigmoidoscope:	
Median 7.0 cm (range, 0–12.0 cm)	
**Location**	**Number of patients (%)**
0–5 cm	12 (26)
>5–10 cm	30 (65)
>10–12 cm	4 (9)
	
**Local stage (MRI)**	**Number of patients (%)**
T3	38 (83)
T4	8 (17)
N0	10 (22)
N1	26 (56)
N2	10 (22)
	
*Relation of primary tumour to mesorectum (MRI)*	
Mesorectal fascia not involved^a^	4
Mesorectal fascia potentially involved (⩽1 mm from primary tumour) (*n*)	15
Mesorectal fascia involved but not breached by primary tumour) (*n*)	12
Mesorectal fascia breached (*n*)	15

a Low third cancers ⩽50 mm from anal verge, one with definite involvement of external anal sphincter.

**Table 2:** Worst toxicity grade and incidence according to dose levels during chemoradiation and up to 4 weeks afterwards (numbers of patients for each toxicity level shown)

	**Dose level**																		
	**1 *n*=3**					**2 *n*=6+8=14**					**3 *n*=7+16=23**					**4 *n*=6**			
Toxicity grade NCI CTC	1	2	3	4		1	2	3	4		1	2	3	4		1	2	3	4
																			
*Haematologic*																			
Anaemia		2				4	3					5					1		
Leukopenia						1	3	1					3				2		
Thrombopenia												1							
Neutropenia	1					1	3	1					3				1	1	
Haemorrhage	1											1							
																			
*Infective*																			
Non-neutropenic fever/infection (ANC⩾1.0 × 10^9−1^)	1						3					1	1			1			
Neutropenic fever/infection (ANC<1.0 × 10^9^ l^−1^)							1						1					1	
																			
*Biochemical*																			
Hyperbilirubinaemia	1					2					7						1		
AST/ALT						4						3						1	
Creatinine elevation						1						1							
																			
*Gastrointestinal*																			
Stomatitis	2					4						1							
Nausea/vomiting		2				2	3						2				1		
Diarrhoea		2				2	4	3					6					1	
Cholinergic syndrome											3						1		
																			
*Constitutional*																			
Fatigue/lethargy		2				4	6						3				2		
Anorexia	2					3	2						2				1		
Weight loss						2	1					4					1		
																			
*Dermatology*																			
Palmar plantar erythrodysesthesia (PPE)	1					1	2						1			1			
Alopecia											4						2		
Skin (radiation dermatitis)		1				3	2					2				2			
																			
*Cardiac*																			
Hypertension													1						
Hypotension	1					2													
Cardiac function											1								

**Table 3:** Number of patients at each dose level who experience the specified number of dose-limiting toxicities

	**Dose level**			
**Number of DLTs per patient**	**1 *n*=3**	**2 *n*=6+8=14**	**3 *n*=7+16=23**	**4 *n*=6**
1	—	3	4	2
2	—	—	3	—
3	—	—	1	—
Total number of patients experiencing at least one DLT	0/3	3/14	8/23	2/6

**Table 4:** Dose intensity: Mean percentage of intended dose of radiotherapy, capecitabine and irinotecan that was delivered at each dose level

	**Dose level 1**	**Dose level 2**	**Dose level 3**	**Dose level 4**
Patients per cohort	3	14	23	6
RT completed (%)	100	100	96	96
Irinotecan completed (%)	100	96	94	93
Capecitabine completed (%)	99	91	82	82

**Table 5:** Pre-treatment MRI scan stage compared with post-chemoradiation MRI stage *n*=43

		**Post-chemoradiation MRI stage**					
		**T0**	**T1**	**T2**	**T3**	**T4**	**Total (%)**
	**T3**	3	0	13	20	0	36 (84)
	**T4**	0	0	0	2	5	7 (16)
	**Total (%)**	3 (7)	0	13 (30)	22 (51)	5 (12)	43 (100)
**Pre-treatment MRI stage**							
		**N0**	**N1**	**N2**	**Total (%)**		
	**N0**	9	0	0	9 (21)		
	**N1**	23	2	0	25 (58)		
	**N2**	6	1	2	9 (21)		
	**Total (%)**	38 (88)	3 (7)	2 (5)	43 (100)		

**Table 6:** Immediate post-operative complications (within 30 days of surgery) within the 40 patients undergoing resection and alive post operatively

**Post-operative complication**	**Number of patients**
Pelvic infection	4 (one with peritonitis also)
Wound infection	3
Serious infection elsewhere	2 (chest, urinary)
Anastomotic dehiscence	2
Perineal wound dehiscence	2
Re-catheterisation necessary	1
Haemorrhage within the operative field necessitating return to theatre	0
Venous thromboembolic event	0
Myocardial infarction	0
Cerebrovascular accident	0
Ventilation needed for >24 h postop	0
Acute respiratory distress syndrome	0
Death	0
Median time spent on ITU/HDU post op in days (range)	0 (0–14)
Readmission necessary after discharge	5^a^
Other serious post-operative complications	4: (Atrial fribrillation) (Enteral feeding required) (Prolonged rectal drain) (Neurogenic bladder)

a One patient readmitted for femoral distal bypass following Hartmann's procedure, probably unrelated to rectal cancer treatment.

**Table 7:** Pre-treatment MRI scan stage compared to histology of the resected specimen (*n*=41)

		**Histology of resected specimen**					
		**T0**	**T1**	**T2**	**T3**	**T4**	**Total (%)**
	**T3**	9	1	4	20	1	35 (85)
	**T4**	2	1	0	3	0	6 (15)
	**Total (%)**	11 (27)	2 (5)	4 (10)	23 (56)	1 (2)	41 (100)
**Pre-treatment MRI stage**							
		**N0**	**N1**	**N2**	**Total (%)**		
	**N0**	9	0	0	9 (22)		
	**N1**	20	4	0	24 (59)		
	**N2**	4	1	3	8 (20)		
	**Total (%)**	33 (80)	5 (12)	3 (7)	41 (100)		

**Table 8:** Phase I/II studies of neoadjuvant rectal cancer CRT using concurrent capecitabine plus irinotecan

**Author**	**Phase**	**Total no. of subjects**	**Pelvic staging method**	**Clinical stage**	**RT dose**	**Capecitabine dose**	**Irinotecan dose**	**Grade 3/4 toxicity**	**PCR rate**	**R0 resection rate**
Hofheinz *et al* (2005)	I dose escalation	19	TRUS Pelvic CT	T3 *n*=18 T4 *n*=1	50.4 Gy 28 Fr 5.5 weeks	500 mg m^−2^ bd days 1–38^a^	50 mg/m^2^ weekly × 5	1/12 gr 3 fatigue	4 of 19 resected =21%	NS
						625 mg m^−2^ bd days 1–38	50 mg/m^2^ weekly × 5	3/7 gr 3 diarrhoea 1/7 gr 3 N+V 1/7 gr 3 anorexia 1/7 gr 3 leukopenia		
Klautke *et al* (2006)	I/II	28	TRUS Pelvic CT	T2 *n*=2 T3 *n*=18 T4 *n*=8	55.8 Gy 31 Fr 6 weeks	500 mg m^−2^ bd days 1–43	40 mg/m^2^ weekly × 6	0/3	4 of 25 resected =15%	24/25 (96%)
						650 mg m^−2^ bd days 1–43	40 mg/m^2^ weekly × 6	0/3		
						750 mg m^−2^ bd days 1–43^a^	40 mg/m^2^ weekly × 6	6/16 (38%) gr 3 diarrhoea		
						825 mg m^−2^ bd days 1–43	40 mg/m^2^ weekly × 6	3/6 gr 3 diarrhoea 2/6 gr 4 diarrhoea 1/6 gr 3 HFS 1/6 gr 3 leukopenia		
Willeke *et al* (2007)	II	36	TRUS Pelvic CT	T2 *n*=4 T3 *n*=26 T4 *n*=5	50.4 Gy 28 Fr 5.5 weeks	500 mg m^−2^ bd days 1–38	50 mg/m^2^ weekly × 5	4/36 (11%) gr 3 diarrhoea 2/36 gr 3 N+V 1/36 gr 3 fatigue 7/36 gr 3 leukopenia 2/36 gr 4 leukopenia	5 of 34 resected =15%	34/34 100%
Klautke *et al* (2007)	II	20	TRUS Pelvic CT	T3 *n*=18 T4 *n*=2	55.8 Gy 31 Fr 6 weeks	750 mg m^−2^ bd days 1–14, 22–35	50 mg/m^2^ weekly × 4 (days 1, 8, 22, 29	2/10 (10%) gr 3 diarrhoea	0	20/20 100%
	II	20		T2 *n*=1 T3 *n*=16 T4 *n*=3		750 mg m^−2^ bd days 1–14, 22–35	60 mg/m^2^ weekly × 4 (days 1, 8, 22, 29	3/20 (10%) gr 3 diarrhoea	7 of 20 resected =35%	19/20 (95%)

Abbreviations: Day 1=first day of RT; TRUS=trans rectal ultrasound; N+V=nausea and vomiting; NS=not stated; HFS=hand foot syndrome.

a Recommended dose.
